# Nuclear envelope and chromatin choreography direct cellular differentiation

**DOI:** 10.1080/19491034.2024.2449520

**Published:** 2025-02-12

**Authors:** Anjitha Nair, Jayati Khanna, Jashan Kler, Rohith Ragesh, Kundan Sengupta

**Affiliations:** Chromosome Biology Lab (CBL), Indian Institute of Science Education and Research (IISER) Pune, Maharashtra, India

**Keywords:** Cell differentiation, chromatin organization, LADs, LEM, NPC, nuclear architecture, nuclear lamina, nuclear mechanobiology, Nuclear Pore Complex

## Abstract

The nuclear envelope plays an indispensable role in the spatiotemporal organization of chromatin and transcriptional regulation during the intricate process of cell differentiation. This review outlines the distinct regulatory networks between nuclear envelope proteins, transcription factors and epigenetic modifications in controlling the expression of cell lineage-specific genes during differentiation. Nuclear lamina with its associated nuclear envelope proteins organize heterochromatin via Lamina-Associated Domains (LADs), proximal to the nuclear periphery. Since nuclear lamina is mechanosensitive, we critically examine the impact of extracellular forces on differentiation outcomes. The nuclear envelope is spanned by nuclear pore complexes which, in addition to their central role in transport, are associated with chromatin organization. Furthermore, mutations in the nuclear envelope proteins disrupt differentiation, resulting in developmental disorders. Investigating the underlying nuclear envelope controlled regulatory mechanisms of chromatin remodelling during lineage commitment will accelerate our fundamental understanding of developmental biology and regenerative medicine.

## Introduction

Cell differentiation and lineage commitment are complex and well-orchestrated biological processes. The stages of cell differentiation are coordinated by crosstalk between various levels of chromatin organization, epigenetic modifications, and transcriptional regulation, resulting in appropriate and accurate gene expression programs. Diverse cell types are generated from a single genome as a result of differential chromatin organization and timely expression of lineage-specific genes. In the interphase nucleus, each chromosome occupies a unique and specific Chromosome Territory (CT) [1]. The chromatin within the CTs is organized into Topologically Associating Domains (TADs) and further into A and B chromatin sub-compartments. Compartment A is organized towards the nuclear interior and consists of gene-rich, transcriptionally active euchromatin enriched in active histone marks. Conversely, compartment B, closer to the nuclear periphery, is gene-poor, transcriptionally repressed heterochromatin and enriched in inactive histone marks [2]. Furthermore, chromatin interacts with nuclear envelope proteins at the nuclear periphery to regulate spatio-temporal organization and transcription during differentiation and development.

**Figure 1.**
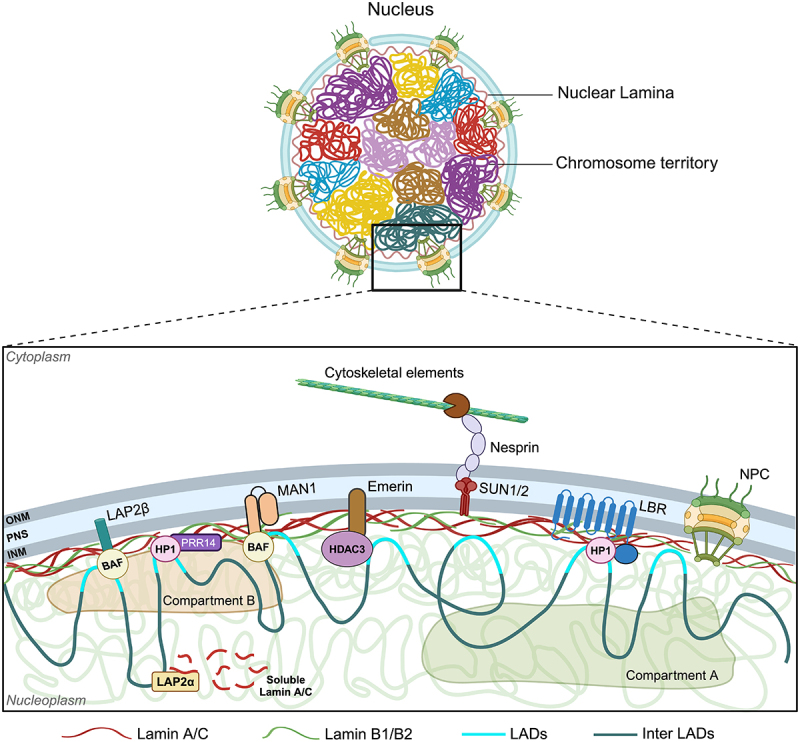
**Role of nuclear envelope proteins in chromatin organization.** The nuclear membrane is composed of the Outer Nuclear Membrane (ONM) and Inner Nuclear Membrane (INM), separated by a PeriNuclear Space (PNS). Nuclear lamina is formed by a filamentous meshwork of A- and B-type lamins along with associated LEM-domain containing proteins (LAP2, emerin, and MAN1), and the Lamin B Receptor (LBR), which spans the INM and is interspersed by Nuclear Pore Complex (NPC). Phosphorylated A-type lamins are localized in the nucleoplasm. The Linker of Nucleoskeleton and Cytoskeleton (LINC) complex is composed of Nesprin and SUN, which are responsible for force transmission from cytoskeletal elements to the nucleus. This schematic depicts the interaction of nuclear envelope proteins with chromatin. Chromatin associated with the nuclear lamina is referred to as Lamina-Associated Domains (LADs). Spatial organization of LADs is maintained at the nuclear envelope by direct interaction of chromatin to nuclear lamina and via chromatin-binding proteins such as Heterochromatin Protein 1 (HP1), Barrier to Autointegration Factor (BAF), and Histone Deacetylase 3 (HDAC3). Soluble lamin A/C can interact with chromatin via LAP2α in the nuclear interior. LADs are rich in heterochromatin and reside in compartment B, while euchromatin is localized in compartment A (adapted from [7]). (created with BioRender.com).

The nuclear envelope is a double lipid bilayer membrane enclosing the nucleus that contains the genome and other sub-nuclear components. The nuclear envelope consists of an Outer Nuclear Membrane (ONM) and an Inner Nuclear Membrane (INM) separated by a PeriNuclear Space (PNS). Embedded through the nuclear envelope are the Nuclear Pore Complexes (NPCs) that regulate transport across the nuclear envelope [3]. Proteomics analysis reveals 148 proteins enriched at the nuclear envelope, such as lamins, lamin-interacting proteins and Nuclear Envelope Transmembrane (NET) proteins, among others [4,5]. The INM is underlined by a fibrous meshwork of nuclear lamina composed of A- and B-type lamins. Regions of chromatin that interact with the nuclear lamina are referred to as Lamina-Associated Domains (LADs), which are largely transcriptionally inactive heterochromatin [6]. Transmembrane proteins including Lamin B-Receptor (LBR) and LEM-domain proteins – LAP1, LAP2, Emerin, MAN1 and LEMD2 interact with lamins (Figure 1). The LINC complex (consisting of nesprins and SUN proteins) and lamins establish the physical coupling between the nucleoskeleton and cytoskeleton that enables the nucleus to respond to mechanical signals from the extracellular microenvironment [8]. Nuclear envelope proteins regulate nuclear structure, chromatin organization and gene expression. Here, we provide a comprehensive review of the mechanisms by which nuclear envelope proteins regulate cell differentiation in concert with chromatin organization and transcriptional regulation. We also discuss how pathogenic mutations of the nuclear envelope encoding genes manifest as abnormal differentiation and development.

## Nuclear lamins

Lamins are type V intermediate filament proteins ubiquitously expressed across all differentiated mammalian cells [9,10]. Lamins are primarily of two subtypes: A- and B-type lamins. In mammalian somatic cells, A-type lamins are encoded by the *LMNA* gene and express two major alternatively spliced isoforms, lamin A and C; B-type lamins are further classified into lamin B1 and B2, encoded by *LMNB1* and *LMNB2* genes, respectively [11]. Lamins are composed of a globular head (N-terminal), central rod (α-helical coiled-coil domain), and globular tail domain (C-terminal) [12,13]. Lamin monomers assemble into coiled-coil parallel dimers and further into antiparallel head-to-tail polymers to form a complex filamentous network [14]. In the interphase nucleus, A-type lamins are predominantly localized at the nuclear envelope, but also in the nucleoplasm, whereas B-type lamins are primarily localized at the nuclear envelope [15]. Cryo-electron tomography data reveals that the lamin meshwork is composed of globular structures along the filaments, imparting a fibrillar network-like organization. Super-resolution imaging reveals spatial segregation of the A- and B-type lamins within the lamin meshwork [14]. Such a remarkable structural and architectural diversity of the nuclear lamins have a distinct bearing on its functional diversity, ranging from nuclear and chromatin organization, mechanotransduction, DNA damage repair, and transcriptional regulation, among others, especially in the context of development and differentiation, which involves major changes in cellular and nuclear architecture. The stoichiometry of the A:B type lamins varies between progenitor and differentiated cells across cell types. Differentiated cells have relatively higher expression of A-type lamins than pluripotent cells. B-type lamins are expressed throughout development [16]. However, during neuronal differentiation, lamin B1 levels are relatively reduced in mature neurons compared to progenitor cells [17]. Altered or mutant lamins in progenitor cells impair differentiation and development [18–20].

## Overview of Lamina-Associated Domains (LADs)

The nuclear lamina plays a critical role in regulating higher-order spatial and temporal organization of the genome. DamID has been used to identify LADs – regions of the genome that are closely associated with the lamins [6]. The length of LADs ranges from ~0.1 to 10 Mb and constitute ~30–40% of the genome [21]. LADs are largely composed of A-T rich and gene-poor regions of the genome, which are typically transcriptionally inactive [22]. LADs are enriched in constitutive (H3K9me2/me3) and facultative (H3K27me3) heterochromatin marks [21,23]. The protein PRR14 tethers heterochromatin enriched in H3K9me3 to the nuclear lamina [24].

LADs are further classified into constitutive LADs (cLADs) and facultative or variable LADs (fLADs or vLADs). cLADs are composed of gene-poor regions that are constitutively associated with the nuclear lamina across different cell types. Genome-wide DamID analysis of lamin B1 reveals that ~71% of the LADs are conserved across murine cell types: Embryonic Stem Cells (ESCs), neural precursor cells, astrocytes, and Mouse Embryonic Fibroblasts (MEFs). LADs are also highly conserved across species, especially between murine and human ESCs [22]. Conversely, fLADs or vLADs are genomic regions whose association with the lamina is specific to cell type and stage of development [25–27]. A combination of Hi-C (captures genome-wide intra- and inter-chromatin interaction) and DamID (profiling of protein-chromatin interactions) analyses reveal that LADs are largely enriched in compartment B of chromatin [28]. Inter-LADs (iLADs) are regions between the LADs that are positioned relatively away from the nuclear lamina. Interestingly, the iLADs are >10 kb away from the LADs in compartment A, relatively more gene-dense than LADs, and transcriptionally active [21,28,29]. The insulator protein CTCF is enriched ~5–10 kb outside of LADs, suggesting that CTCF is involved in marking LAD borders and facilitates chromatin looping with distal CTCFs in inter-LADs [21]. DNA in the iLADs can loop away from the nuclear lamina to associate with nuclear substructures, including transcription factories formed by the clustering of active RNA Pol II [29,30].

## LAD dynamics during cell lineage commitment

**Figure 2.**
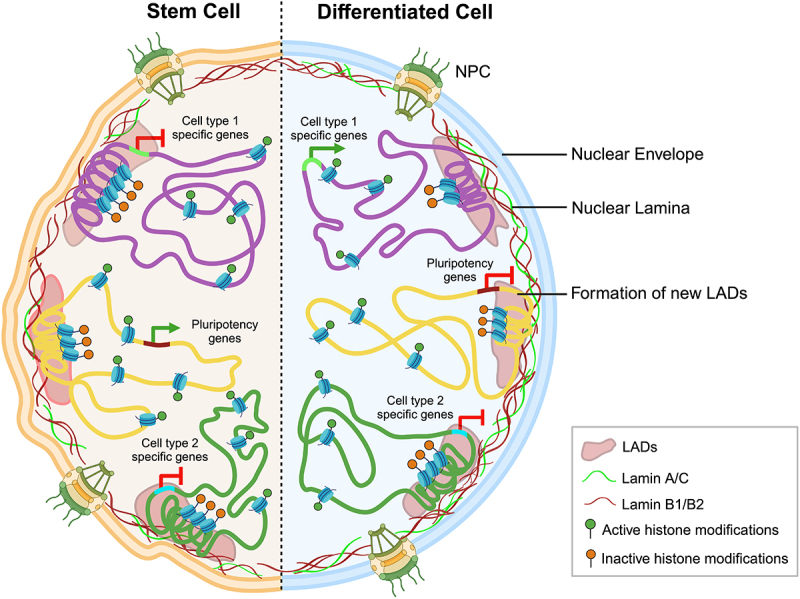
**Schematic view of LAD reorganization upon lineage commitment.** This schematic depicts the spatial organization of pluripotency and lineage-determining genes in stem cells (left) that undergo differentiation into cell type 1 (right). The composition of nuclear lamina is altered during differentiation accompanied by an upregulation of lamin A/C. The developmental genes (cell type 1-specific) dissociate from LADs and are activated during cell-lineage commitment, along with enrichment of active histone marks. Whereas developmental genes specific to other cell types (cell type 2-specific) remain associated with the nuclear lamina. Meanwhile, in stem cells, the active pluripotency genes are localized in the nuclear interior. Upon differentiation, pluripotency genes shift towards the nuclear lamina and are enriched with inactive histone marks, resulting in their repression. Thus, spatial organization of genes with respect to the nuclear lamina may correlate with their transcriptional status [25–27,32,33]. (created with BioRender.com).

The commitment of cells to a specific lineage involves alteration in the spatial organization of many genomic regions [25]. Repositioning of LADs largely contributes in regulating the expression of pluripotency and lineage-specific genes across species, including *C. elegans*, *Drosophila*, mouse and human [25,26,31]. A comprehensive analysis of lamin B1 DamID data, gene expression, histone occupancy maps, and Hi-C datasets across stem cells and differentiated cell types shows that lamins further reinforce a repressive chromatin state during differentiation [26,27]. Trends of ‘locking’ and ‘unlocking’ of pluripotency genes by their association and dissociation from the nuclear lamina are common features of adipogenesis, neurogenesis, cardiomyogenesis, and myogenesis, which are further discussed in detail [25–27,32,33] (Figure 2).

Remarkably, rearrangements of LADs and iLADs occur in a non-stochastic manner during adipogenesis [33,34]. Of note, adipogenic genes, including *MAFB*, *RNASEL*, *DIO2*, and *TRIM32*, detach from the LADs upon adipogenic stimulation (~24 h), devoid of changes in gene expression but are subsequently upregulated (~72 h) in differentiating Adipogenic Stem Cells (ASCs). In contrast, the non-adipogenic genes are topologically restrained in the repressive confines of the LADs. Differentially Expressed (DE) genes are predominantly in euchromatic regions of reduced lamin B1 occupancy during differentiation. In addition, the activation of DE genes in adipocytes also depends on epigenetic pre-priming by active or inactive histone modifications at their enhancers and promoters before differentiation [33]. It is noteworthy that adipogenesis is hampered in ASCs expressing a lipodystrophic mutant of lamin A/C (R482W) [18,19] – a loss of function mutant that increases the expression of an anti-adipogenic microRNA – miR335 [18,19]. ASCs expressing mutant lamin A/C R482W unlocks the *MIR335* gene locus from the nuclear lamina and clusters it with distal enhancers, resulting in its transcriptional activation [19].

The non-random organization of lineage-determining genes is specific to the stage of differentiation and cell fate decision. For example, in *C. elegans*, the transgene *myo-3*, which is involved in muscle contraction, shifts toward the nuclear interior, consistent with its enhanced expression, upon muscle differentiation. Interestingly, the relocalization of the *myo-3* gene locus to the nuclear interior is specific to muscle cells and not observed for other cell lineages, suggesting cell context is a key determinant of gene loci dynamics [31]. During neural differentiation, pluripotency genes – *Nanog, Klf4*, and *Oct4* are spatially proximal to the nuclear lamina with decreased expression. The neuronal-specific gene, *Pcdh9* is unlocked from the nuclear lamina, consistent with its transcriptional activation upon the transition of ESCs to multipotent neural precursor cells and terminally differentiated astrocytes [25]. Upon induction of neural differentiation in ESCs by Retinoic Acid (RA), the *Mash1* gene-encoding transcription factor involved in neural development is transcriptionally activated and relocalized into the nuclear interior [35]. Additionally, temporal repositioning of genes encoding master regulators of development dictates cell-lineage commitment choice. For instance, the gene Myocyte Enhancer Factor 2C (*MEF2C)* is involved in neurogenic and cardiogenic differentiation. *MEF2C* is upregulated and relocalized away from the nuclear lamina at Day 3 in RA-induced neurogenesis in multipotent P19 embryonic carcinoma cells. In contrast, transcriptional upregulation and repositioning of *MEF2C* is observed at Day 6 in DMSO-induced cardiogenesis in P19 cells. However, during early cardiogenic differentiation (Day 3), the cardiac gene *MYOCD* relocalizes from the nuclear periphery to the nuclear interior, as shown by 3D-immuno Fluorescence In Situ Hybridization (FISH) [32]. Another essential gene for cardiac development, *TBX20*, is also reorganized from the LAD in ESCs to non-LAD regions in cardiomyocytes, accompanied by its increased expression [27]. Taken together, these results highlight an overarching role for the nuclear envelope to function as regulatory hub of chromatin organization and gene expression. However, how proteins in the nuclear envelope function to tether and untether chromatin in a highly calibrated manner to facilitate chromatin accessibility for transcription factors remains unclear. We surmise that nuclear envelope proteins and transcription factors collectively modulate chromatin opening and activity. The abrogation of the external cue is likely to re-sequester chromatin back to its endogenous state of compaction and requires experimental validation.

Thus, the association of gene loci with LADs is useful for anticipating gene expression outcomes, which are usually associated with gene repression. However, the gene *SCCPDH*, which encodes for saccharopine dehydrogenase enzyme required in lysine metabolism, is an exception. *SCCPDH* shows high lamin B1 occupancy in both ESCs and differentiated cardiomyocytes; nevertheless, its expression is elevated in cardiomyocytes [27]. While there is strong evidence correlating LAD association with gene repression, alternate mechanisms potentially regulate spatial organization and transcriptional activity of gene loci in the nucleus. Comparison between ChIP-Seq and DamID data sets of lamin B1 and H3K9me2 occupancy between human ESCs and in vitro differentiation of 11 ESC-derived cells suggests that genes with an intermediate enrichment of lamin B1 and H3K9me2 display dynamic association with LADs during differentiation. Therefore, lamin occupancy levels (high or intermediate) can regulate the activation or inactivation of lineage-specific genes during differentiation [27]. We speculate that cell-fate determining genes within LADs are poised, facilitating (i) activation during differentiation and (ii) switching from the inactive compartment B to the more active compartment A. Furthermore, analysis of multi-omics datasets across cell types and cell states is likely to provide further insights about the cause-and-effect relationship between the association of gene loci with LADs and their gene expression levels.

## Chromatin remodelers facilitate chromatin tethering at the nuclear envelope

Although nuclear lamins directly interact and tether chromatin to the nuclear envelope, chromatin readers and writers are also involved in the maintenance of the chromatin states of LADs. Chromatin plasticity is essential for maintaining pluripotency in ESCs. Upon ESC differentiation, chromatin plasticity is reduced by the downregulation of histone acetylation and elevation of H3K9 methylation and lamin A/C levels that commit cells to specific lineages [36]. Histone-lysine N-methyltransferase, G9a deposits H3K9me2 and maintains pluripotency factors *Sox6*, *Itga2b*, *Gata1*, and *Fgf3* in a silenced state to facilitate hematopoietic stem cell differentiation [37]. Consistently, G9a also silences *Oct4* and *Rex1* in somatic cells by depositing H3K9 methylation and prevents reprogramming of somatic cells to iPSCs (induced Pluripotent Stem Cells) [38]. Subsequently, Heterochromatin Protein 1, HP1-α, β, and γ bind to H3K9-methylated promoters of *Oct3/4* [39]. Additionally, HP1α-associated heterochromatin also organizes into phase-separated droplets [40,41]. Since HP1α tethers heterochromatin with the nuclear lamina, phase separation functions as a complementary mechanism for transcriptional repression by chromatin compaction [42,43]. However, the role of phase separation of HP1α and its functional consequences in the context of differentiation remains unexplored. Moreover, chromatin compaction is also maintained by levels of histone acetylation, which is regulated by a balance between Histone Deacetylases (HDACs) and Histone Acetyltransferases (HATs). HDACs deacetylate the histones to neutralize positively charged lysine in histone tails which result in chromatin compaction and gene repression [44]. Treatment of mouse and human ESCs with HDAC inhibitors (HDACi) maintains ESCs in an undifferentiated state [45]. Interestingly, HDAC3, independent of its deacetylase activity, tethers cardiac genes at the nuclear lamina in ESCs, inhibiting cardiogenesis and facilitating myogenesis [46]. In summary, lamins function in close coordination with chromatin remodelers to regulate chromatin organization and dynamics during differentiation.

## Mechanoregulation of differentiation

The transmission of mechanical forces from the cytoskeleton to the nuclear lamina is mediated by the LInker of Nucleoskeleton and Cytoskeleton (LINC) complex. LINC is a protein complex that spans across the nuclear envelope and is composed of nesprin at the ONM and SUN at the INM (Figure 1). Nesprin at the cytoplasmic face interacts with cytoskeletal proteins, including actin, microtubules, and intermediate filaments. The KASH domain of nesprin extends into the perinuclear space to interact with the SUN domain. On the nucleoplasmic side, SUN1 and SUN2 interact with lamin A/C [8,47–50]. The LINC-lamin axis functions as a key protein machinery for the transmission of forces across the nuclear envelope. During keratinocyte differentiation, the LINC complex inhibits the differentiation of epidermal stem cells by impeding the relocalization of Epidermal Differentiation Complex (EDC) genes toward the nuclear interior. Transcriptional activation of EDC genes by their release from the nuclear lamina is essential for keratinocyte differentiation. Depletion of both SUN1 and SUN2 disrupts force transmission across the LINC-lamin axis, impacting the chromatin compartmentalization of EDC genes with respect to the nuclear lamina [51]. This direct evidence shows how mechanical forces regulate chromatin organization during differentiation.

A-type lamins are mechanosensitive and respond to mechanical forces by dynamically altering their levels and sub-nuclear localization at the nuclear periphery and nucleoplasm [52]. These coordinated changes in lamin A/C in response to forces from the external microenvironment regulate cell fate determination of Mesenchymal Stem Cells (MSCs) [52,53]. The stiffness of the extracellular matrix regulates the differentiation of MSCs into specific lineages. Stiffer substrates prime MSCs for osteogenic differentiation, while softer substrates favor adipogenic differentiation. Both cell lineage specifications are regulated by levels of lamin A/C [52,54]. Increased cytoskeletal tension in cells on stiffer substrates stabilizes lamin A/C at the nuclear envelope by significantly attenuating lamin A/C phosphorylation. While overexpression of lamin A/C in MSCs induces osteogenesis, knockdown of lamin A/C leads to adipogenesis [52]. Lamin A/C regulates differentiation of MSCs via its crosstalk with mechanosensitive signaling pathways, including RA, WNT/β-catenin signaling, YAP/TAZ, and MKL1-SRF signaling pathways as described in detail below [52,54–56].

Lamin A/C operates in a feedback loop with RARG, which belongs to the nuclear receptor superfamily of Retinoic Acid Receptors (RAR) [52]. RARG participates in MSC differentiation by directly regulating lamin A/C gene expression through its RA-Responsive Elements (RARE) binding sites on the *LMNA* promoter. The RA pathway functions as a downstream effector of substrate stiffness. On stiffer substrates, the addition of an antagonist of RARG enhances lamin A/C expression, resulting in osteogenesis. Interestingly, higher levels of lamin A/C promote nuclear translocation of RARG. In contrast, depletion of lamin A/C impedes MSC osteogenesis initiated by the antagonist of RARG, consistent with more cytoplasmic localization of RARG [57]. Thus, the lamin A/C-RARG feedback loop responds to substrate stiffness to guide MSC differentiation.

Lamin A/C also promotes MSC osteogenesis by regulating the nuclear import and export of mechanosensitive transcription factors and co-factors in cells on stiffer substrates. Overexpression of lamin A/C in cells cultured on softer substrates abrogates nuclear translocation of early adipogenic transcription factor, Sterol Regulatory Element Binding Protein 1 (SREBP1) [52]. Elevated levels of lamin A/C activate the WNT/β-catenin pathway by promoting nuclear translocation of β-catenin via emerin [56,58]. Nuclear translocation of β-catenin is restricted by its interaction with emerin in the cytoplasm. The localization of emerin at the nuclear envelope is maintained through its association with lamin A/C. On softer substrates when lamin A/C levels are low, emerin partly mislocalizes into the cytoplasm, where it binds to β-catenin to curtail its re-entry into the nucleus [58]. However, on stiffer substrates, β-catenin translocates into the nucleus and binds to Lef-1, which occupies the Transcription Start Site (TSS) of the osteogenic transcription factors *RUNX2* and *OSX* [54]. Furthermore, an alternate force paradigm of constraining hESCs into different geometrically defined ECM induces mesoderm specification by regulating adhesion-tension-dependent release of β-catenin from E-cadherin to activate Wnt ligand expression [59].

Megakaryoblastic Leukemia-1 (MKL1, also termed MRTFA/myocardin-related transcription factor A) is another mechanosensitive transcriptional co-activator involved in differentiation and cell fate reprogramming. MKL1 nuclear import is regulated by actin polymerization. MKL1 is bound to G-actin, preventing its nuclear translocation [60,61]. Actin polymerization induced by mechanical stimuli such as cyclic strain, substrate micropatterning, and increased substrate stiffness releases MKL1 from G-actin and thereby MKL1 is imported into the nucleus. Lamin A/C expression and nuclear translocation of MKL1 are positively correlated, i.e., *LMNA* knockdown cells show lower nuclear levels of MKL1, as opposed to *LMNA* overexpressing cells having higher nuclear levels of MKL1 [62–64]. Nuclear accumulation of MKL1 subsequently activates the SRF pathway and downstream genes involved in modulating actin dynamics [60,61]. The MKL1-SRF pathway promotes osteogenesis in a lamin A/C-dependent manner in response to cyclic strain [64]. In contrast, the MKL1-SRF pathway inhibits adipogenesis, working antagonistically with the regulator of adipogenesis – Peroxisome Proliferator-Activated Receptor gamma (PPARγ) [65]. Moreover, the LINC complex restricts the reprogramming of somatic cells by inhibiting MKL1 activity [66].

Another extensively studied mechanosensitive pathway is the YAP/TAZ pathway. Nuclear import and expression of YAP/TAZ increases on stiffer substrates. YAP/TAZ associates with the transcription factor TEAD to promote osteogenesis of MSCs on stiffer substrates [67,68]. Mechanical load on mesenchymal progenitor cells also activates the YAP/TAZ pathway, which is crucial for MSC proliferation and self-renewal [69]. The nuclear translocation of YAP is dependent upon the transduction of contractile forces. Nuclear compression is controlled by tension across the LINC complex, and nuclear localization of YAP is reduced upon the disruption of the LINC complex [70]. LINC complex-lamin A/C coupling controls the stretching of the NPC, which regulates the nuclear import of YAP [71,72]. Independent lines of evidence suggest a crosstalk between lamin A/C and nuclear localization of YAP/TAZ [20,52,72,73]. However, there are contradictory results regarding the regulation of YAP/TAZ by lamin A/C across cell types, cell state, and the type of force applied. For instance, the depletion of *LMNA* decreases YAP1 protein level, although the transcript levels and nuclear localization are unaffected. However, overexpression of *LMNA* also decreases YAP1 levels and its nuclear localization on stiffer substrates [52]. Relatively higher levels of lamin A/C increase nuclear rigidity, which potentially attenuate the nuclear import of YAP1 through NPC [74]. Impaired mechanosensing responses due to *LMNA* mutation ΔK32 or Nesprin1?KASH mutants show persistent nuclear translocation of YAP and are associated with congenital myopathy [20,73]. In summary, extensive studies across diverse cell types and model systems will unravel the complexity of regulatory networks between lamin A/C and YAP/TAZ signaling.

Collectively, lamin A/C is a critical structural and functional regulator of differentiation. Lamins maintain cell-lineage commitment by (i) sensing and transducing mechanical cues from the microenvironment (ii) interacting with signaling pathways (iii) modulating nuclear translocations of transcription factors and (iv) regulating 3D spatial chromatin organization and transcriptional regulation. However, the role of LINC complex and lamin A:B stoichiometry in mechanoregulation of differentiation awaits further investigation. It remains unclear how alterations in mechanical forces such as stiffness, compression, and strain can affect the organization of LADs and chromatin compartmentalization. Mechanical signals alter the tension at the nuclear envelope, resulting in changes in the organization of lamins and potentially its interactors. For instance, in cells cultured on softer substrates, localization of lamin A/C increases in the nucleoplasm owing to the phosphorylation of nuclear lamins [75]. Therefore, the altered localization of lamin A/C changes its interaction with chromatin at the nuclear periphery and interior [76]. We surmise that DamID based approaches with relatively stable nuclear envelope proteins as anchors, in conjunction with high-resolution Fluorescence In Situ Hybridization (FISH), is likely to unravel the effect of rapid mechanical stimuli regulating lamina-chromatin interactions and spatial positioning of lineage-determining genes during differentiation.

## Lamin B Receptor (LBR)

LBR is a 615 aa long INM protein with a basic N-terminal domain that interacts with B-type lamins and a TUDOR domain that binds to DNA [77]. LBR is involved in chromatin organization, regulation of gene expression, and protection of genome from chromosomal instability [78,79]. LBR facilitates the association of the nuclear envelope with heterochromatin, while its loss de-represses heterochromatin in LADs [80]. The role of LBR in differentiation is unclear. However, LBR knockdown dysregulates pluripotency and differentiation. Loss of both LBR and lamin A/C shows an inversion of the conventional nuclear architecture, leading to the internalization of heterochromatin. The expression of lamin A/C succeeds LBR expression during early stages of development. This temporal coordination between LBR and lamin A/C expression is a key step required for differentiation [79].

While LBR is predominantly localized in the nucleus, LBR shuttles to the cytoplasm in differentiated germ layer cells (endoderm and mesoderm) during gastrulation. The mislocalization of LBR in mesoderm cells increases lamin B1 dynamics, attributing LBR as an anchor for lamin B1. LBR knockdown in iPSCs upregulates expression of the differentiation marker *HNF-3β* [81]. In addition, LBR expression in the basal layer of the epidermis is a prerequisite for normal skin differentiation. Epidermal hypoplasia, characterized by downregulated keratin 10, hampers keratinocyte differentiation accompanying LBR overexpression. Mutant lamin A/C is associated with Hutchinson-Gilford Progeria Syndrome (HGPS), which shows similar hallmarks of impaired keratinocyte differentiation and upregulated LBR expression [82]. Taken together, these results strongly implicate LBR in maintaining heterochromatin at the nuclear envelope and a key regulator of gene expression during differentiation.

## Nuclear Envelope Transmembrane (NET) proteins

NETs are a large group of proteins embedded in the nuclear envelope that interact with the cytoskeleton at the ONM and with the nuclear lamina and chromatin at the INM. NETs play a critical role in maintaining nuclear structure, signal transduction across the nuclear envelope and regulation of chromatin organization and gene expression. A few NETs have been reported to be involved in cell differentiation. Due to tissue-specific expression of NETs, they are likely to promote differentiation of distinct cell lineages [83]. For example, NET9, NET25, NET32, NET37 and NET39 are upregulated during muscle differentiation, while TMEM120A (NET29) and TMEM120B are upregulated during adipocyte differentiation [84,85]. NETs directly interact with chromatin to regulate the spatial organization of genes and chromosome territories. NET29, NET39 and NET47 facilitate localization of chromosome 5 territory proximal to the nuclear periphery in human HT1080 fibroblast cells [86]. NET39 also repositions chromosome 8 territory from the nucleoplasm to the nuclear periphery upon myogenic differentiation. During myogenesis, muscle-specific NETs – NET39, Tmem38A, and WFS1 promote localization of critical muscle genes to the nuclear periphery, causing their repression [87]. Interestingly, during adipogenesis, Tmem120a association with chromatin represses muscle-specific genes and promotes the expression of adipogenic genes. This was displayed in Tmem120a^-/-^ mice where adipogenic genes are repressed and myogenic genes are derepressed [88]. Thus, NETs provide an additional layer for regulation of genome organization at the nuclear periphery. However, the precise molecular mechanism by which NETs modulate differentiation across tissue types require further investigation. A possible approach could be identifying NETs that are upregulated during tissue-specific differentiation followed by their DamID analysis and RNA-Seq upon NET knockdown to identify a correlation between NET lineage-specific genes and their expression profiles.

## LEM-domain proteins

The LEM-domain proteins share a common LEM domain that was initially identified in Lamina-Associated Polypeptide 1 and 2 (LAP1 and LAP2), Emerin, and MAN1 [89]. Seven independent genes in the mammalian genome encode for LEM-domain proteins: *LAP2, Emerin, MAN1, LEM2, LEMD1, Ankle1*, and *Ankle2* [90]. LEM-domain proteins are primarily localized in the INM and interact with the nuclear lamins. Some of these proteins are distributed in the ER (Ankle 2) and the nuclear interior (LAP2α, Ankle 1), suggesting a novel mode of inter-organellar crosstalk mediated by these proteins [91]. LEM-domain proteins primarily regulate nuclear structure, nuclear lamina assembly, chromatin organization, and gene regulation [92–94]. LEM-domain proteins show functional redundancy; for instance, LAP2 (β, γ, δ, and ε) compensates for emerin deletion, and emerin compensates for MAN1 deficiency [79,95]. This is potentially due to their shared protein interactomes [96]. The globular LEM domain is a bi-helical motif consisting of ~40 amino acids that interact with the chromatin-associated protein Barrier-to-Autointegration Factor (BAF) [97–99]. Mutation of emerin or depletion of BAF inhibits differentiation and causes loss of primordial germ cells in *Drosophila*. BAF is mislocalized from the nuclear lamina in emerin mutants, suggesting the significance of emerin-BAF interaction for differentiation [100].

Expression of LEM-domain proteins is dramatically altered during differentiation, for instance, during spermiogenesis, wherein the spermatid differentiates into mature spermatozoa. In human spermatids – LEMD1, LEMD2, ANKLE2, and LAP2β are present but mislocalized from the nuclear envelope, whereas emerin, LBR, and LEMD3 are absent. Interestingly, all LEM-domain proteins are drastically depleted in mature spermatozoa. This suggests that the nuclear lamina acquires flexibility to facilitate chromatin remodeling for spermiogenesis [106]. We further discuss the specific roles of each LEM-domain protein in differentiation.

LAP2 is a protein encoded by the mammalian *LAP2 (TMPO)* gene. Alternative mRNA splicing results in 9 isoforms of LAP2 in mammals, with a constant N-terminal region (residues 1–168) [101]. The three major isoforms of LAP2 - LAP2α, β, and γ, are abundant in the nucleus [93,102]. The INM-bound LAP2β and γ isoforms primarily associate with the B-type lamins at the nuclear lamina. In contrast, the non-membrane-bound LAP2α binds to the A-type lamins in the nucleoplasm [102,103]. LAP2 has a LAP2 constant region containing the ‘LEM-like’ domain at its N-terminal and LEM domain at its C-terminal. LAP2 associates with DNA directly through the positively charged ‘LEM-like’ domain and indirectly via interaction of the LEM domain with BAF [98]. LAP2α binds to Retinoblastoma (Rb) protein to inhibit E2F/Rb-dependent target genes that allow cell cycle exit. Rb-dependent cell cycle exit is required for adipocyte and muscle differentiation. Hence, overexpression of LAP2α stalls the G1 to S phase transition, resulting in cell cycle exit required for adipocyte differentiation [104]. However, it remains unclear whether the LAP2α-Rb complex binds to the promoter of E2F target genes or functions as part of a larger regulatory complex. Interestingly, new findings show that LAP2α is enriched on the regulatory elements of myogenic genes to promote their transcriptional activation. LAP2α also positively regulates the activity of the transcriptional activator, MRTF-A [105]. Additionally, differentiation is delayed in LAP2α-deficient murine myoblasts with a concomitant reduction in MyoD, a major myogenic differentiation factor [106]. We surmise that LAP2α directly interacts with MyoD to activate its downstream target genes. Collectively, these studies suggest a role of LAP2α in transcriptional regulation, possibly by facilitating the binding of transcriptional regulators on promoters of its target genes. However, the precise mechanism remains to be elucidated.

In the nucleoplasm, LAP2α and lamin A/C interact dynamically through BAF. LAP2α and lamin A/C independently interact with chromatin but show overlapping chromatin occupancy in proliferating myoblasts [107]. During the early stages of muscle differentiation, occupancy of LAP2α but not lamin A/C increases on myogenic genes to promote their transcriptional activation. However, in the absence of LAP2α, nucleoplasmic lamin A/C is enriched on regulatory elements of myogenic genes, causing their repression and perturbation in myogenesis. This indicates competition between LAP2α and lamin A/C for binding sites in chromatin [108]. It is tempting to speculate that the formation of the LAP2α-lamin A/C complex restricts lamin A/C from binding to chromatin. However, this is not the case because LAP2α mutant lacking lamin A/C binding domain also prevents the interaction of lamin A/C with chromatin [107]. We hypothesize that the competition between LAP2α and lamin A/C for chromatin binding might arise from one of the two mechanisms: (i) presence of LAP2α and lamin A/C as a part of a larger protein complex which can be identified by immunoprecipitation followed by mass spectrometry (IP-MS) or (ii) high affinity of LAP2α-BAF for chromatin than lamin A/C-BAF, which needs to be experimentally validated using in-vitro assays such as Surface Plasmon Resonance (SPR).

LAP1 is encoded by the *TOR1AIP1* gene. Myoblasts lacking LAP1 show aberrant differentiation, with delayed expression of key muscle development proteins, myogenin, MyoD and myosin heavy chains (MyHC) [109]. MyoD overexpression did not rescue normal myogenesis in LAP1B mutant (loss-of-function) myoblasts [110]. Mutations in LAP1 are characterized with abnormal nuclear envelope morphologies, implicated in muscular dystrophy, cardiomyopathy, and multisystemic disease [111].

Emerin interacts with lamin A/C and regulates chromatin dynamics, chromosome territories, and gene expression [94]. In addition, emerin interacts with chromatin remodelers including HDAC and histone methyltransferases – EZH2 and G9a to regulate organization of LADs at the nuclear periphery [112,113]. Emerin knockdown in myoblasts downregulates myogenic genes such as desmin and MyoD, which compromises muscle differentiation. Similar attenuation in differentiation patterns is observed in lamin A/C-deficient myoblasts, demonstrating the necessity of emerin and lamin A/C for the differentiation of skeletal myoblasts [114]. Emerin and HDAC3 interaction is crucial for temporal localization and expression of genes required for myogenesis, such as Myf5, MyoD, and Pax7 at the nuclear lamina. Upon differentiation, the expression of Myf5 and MyoD increases along with relocalization of these genes toward the nuclear interior [112]. Emerin loss in myogenic progenitors mislocalizes Myf5 and MyoD in the nucleoplasm [112]. It is likely that in myogenic progenitors, emerin-HDAC association is involved in sequestering myogenic transcription factors in a poised state, which are released from the nuclear periphery upon differentiation. Signaling pathways such as IGF-1, TGF-β, Wnt, ERK/MAPK, JAK/STAT, VEGF, PDGF, Notch, and FGF are dysregulated in emerin null myogenic progenitors. Deregulation of these pathways delays cell cycle withdrawal and shows latency in myogenic differentiation [115–117]. These studies imply that emerin regulates expression of genes required for cell cycle withdrawal and lineage commitment during muscle differentiation.

MAN1 is a SMAD-interacting protein and therefore is an important mediator of TGF-β signaling [118,119]. TGF-β signaling regulates differentiation and development [120]. MAN1 antagonizes TGF-β/BMP signaling in Xenopus and Drosophila [121,122]. MAN1 deficiency in human MSCs promotes osteogenesis by activation of the TGF-β pathway, independent of lamin A/C. MAN1 knockdown enhances the translocation of activated Smad into the nucleus, wherein Smad complexes bind to the transcription factor RUNX2 to activate osteogenic genes. On the contrary, MSCs with either MAN1 overexpression or knockdown impede adipogenesis, indicating that optimal levels of MAN1 are essential for the differentiation of MSCs into adipocytes [123]. MAN1 disruption in mice using MAN1 mutants fails to bind and inhibit SMAD2/3, resulting in their enhanced nuclear accumulation, causing hyper-activation of the TGF-β pathway. This perturbs differentiation of vascular endothelium, resulting in mice lethality [124]. MAN1 is also involved in organ development during early embryogenesis in Xenopus, and deficiency of MAN1 shows severe morphological defects. Emerin compensates for MAN1 function during organogenesis upon co-injection of emerin mRNA [95]. This suggests that LEM domain proteins have redundant functions during differentiation. In summary, the expression of various LEM-domain proteins regulate cell lineage commitment during differentiation and development. This is mediated through chromatin interaction and regulation of multiple signaling pathways that guide the expression of lineage-specific genes.

## The Nuclear Pore Complex (NPC)

In addition to the proteins associated with the nuclear lamina, the nuclear envelope is interspersed by channels at several locations composed of the structurally conserved Nuclear Pore Complex (NPC) proteins [125]. NPC is an essential conduit between the cytoplasm and the nucleus. Macromolecules with a molecular weight <40 kDa passively diffuse through the NPC, while larger molecules require facilitated transport [3]. The NPC is essential for the selective import and export of proteins, metabolites, ions, and RNA. Besides the canonical role of NPC in transport, it is involved in the regulation of cell cycle progression, development, differentiation, DNA damage repair, transcriptional control, and chromatin organization, among others [126,127]. Furthermore, NPC modulates gene expression by mediating nuclear import or recruitment of transcription factors to gene promoters and direct binding to chromatin [128–130]. Here, we highlight the structural organization of the NPC as a prelude to its overarching role in differentiation.

**Figure 3.**
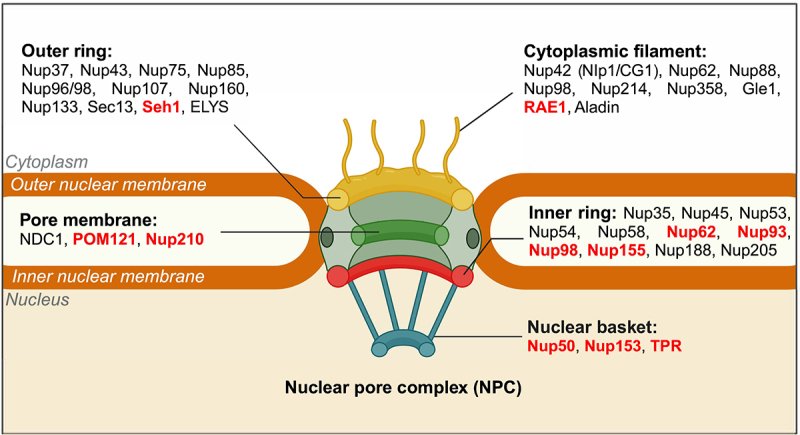
**Structural organization of the Nuclear Pore Complex (NPC).** A schematic representing the composition of the NPC spanning the nuclear envelope in humans. The outer cytoplasmic ring and cytoplasmic filaments of the NPC are shown in yellow. The central transport channel is represented in green. The inner nuclear ring is shown in red and the nuclear basket in blue. Nups in each sub-complex of human NPC are listed. Nups involved in chromatin organization during differentiation are highlighted in red. Seh1 (SEC13 homolog protein 1), ELYS (Embryonic Large molecule derived from Yolk Sac), NDC1 (Nuclear Division Cycle protein 1), POM121 (Pore Membrane Protein of 121 kDa), RAE1 (RNA export 1), Aladin (ALacrima Achalasia aDrenal Insufficiency Neurologic disorder), NLP1 (Nup-Like Protein 1), CG1 (Candidate Gene 1), TPR (Translocated Promoter Region) [133] (created with BioRender.com).

In vertebrates, each NPC spanning the nuclear envelope has multiple copies of ~34 distinct nucleoporin subunits [131]. It is one of the largest protein complexes in the cell, consisting of thousands of nucleoporins (Nups) with an average molecular weight of ~110 MDa [132]. Structurally, NPC has a cylindrical octagonal symmetry, owing to which Nups are present in multiples of eight, i.e., 8, 16, 32, or 48 copies within a single NPC [133]. NPCs are ~120 nm wide and are composed of cytoplasmic filaments, an outer cytoplasmic ring, a central channel, an inner nuclear ring, and a nuclear basket [134]. The central core of the NPC is composed of symmetric nucleoporins forming the inner and outer rings that function as a framework for asymmetric nucleoporins to anchor either at the cytoplasmic or nuclear end to form cytoplasmic filaments and the nuclear basket, respectively (Figure 3) [133].

Nups are classified as on-pore and off-pore Nups. On-pore Nups are strictly localized at the nuclear envelope as a part of the NPC, while off-pore Nups are also localized in the nucleoplasm. However, the experiments and methodologies for categorizing on-pore and off-pore nucleoporins need to be revisited since more high-resolution technologies such as Halo-tags are likely better to resolve the mobility of individual Nups from the NPC. Most nucleoporins have intrinsically disordered regions with FG repeats that form the diffusion barrier and contain binding sites for proteins that aid transport of cargo. On-pore Nups with FG repeats are bound to the NPC at the nuclear envelope [135]. The NPC composition varies in different cell types and is altered in cancers [136–138]. Therefore, studying the stoichiometry of nucleoporins and how they regulate various cellular processes, such as differentiation is required to uncover their multifaceted functions [139]. Here, we discuss the significance of nucleoporins in differentiation and how their dysfunction impacts cell fate decisions.

## Stoichiometry of nucleoporins during differentiation

NPCs exhibit structural plasticity to adapt its architecture and mediate protein trafficking during differentiation. Nucleoporins (such as Nup133, Nup155, and Nup210) are expressed in a cell-type and tissue-specific manner [140,141]. The distinct expression patterns of Nups suggest that they contribute to specialized functions in different tissues. The precise organization and stoichiometry of the nucleoporins within the NPC is essential for embryonic development [142]. Additionally, the composition of NPC is altered during the stages of differentiation. RNA-Seq analysis reveals that most Nups are downregulated during human keratinocyte and murine muscle differentiation [143,144]. During murine muscle differentiation, mRNA levels of Nups present in the outer-ring (Nup107, Nup85, Nup160, Nup43, and Nup37), inner-ring (Nup35, Nup205, Nup188, Nup54, Nup62, and Nup155), pore membrane (Pom121, Ndc1), and nuclear basket of NPC (Nup153 and Nup50) are downregulated. In contrast, the expression of Nup358 and Sec13 is increased, whereas those of Nup214, Nup88, CG1, Nup133, Seh1, Nup93, Gp210, Nup98, and RAE1 are unaltered [144]. While the mRNA levels of TPR are unaltered, its nucleoplasmic pool decreases during muscle differentiation [144,145]. Protein levels of Nup210 and Seh1 are upregulated in muscle and oligodendrocyte differentiation, respectively [146,147]. Nup153 and Nup98 are downregulated during cardiogenesis [148]. Additionally, relatively low levels of Nup153 are detected during neuronal differentiation [149,150]. These stoichiometric changes of Nups coincide with alterations in the nuclear transport of proteins required for transcription and localization of active genes around the NPC, which collectively impact cell differentiation [147,148]. Even the alteration of a single Nup within the NPC affects the differentiation of numerous cell types (Table 1). This highlights that, in addition to its central role in transport, Nups have a non-canonical function in regulating chromatin organization. Variability in Nup expression fine tunes regulatory mechanisms that are crucial during developmental stages and in maintaining tissue homeostasis. Understanding nucleoporin organization in the context of cellular dynamics will not only unravel fundamental biological processes but also provide insights into the mechanisms underlying various diseases.

**Table 1.** Effect of nucleoporin modulation on differentiation.

SI#	Gene	Model system	Perturbation	Effect on differentiation	Reference
**A**	**Pore Membrane Nups**				
1	*NUP210*	Mouse ESCs and C2C12 cell line	shRNA	Disrupts muscle and neural differentiation. Induction of apoptosis without affecting nuclear transport.	[151]
**B**	**Outer ring Nups**				
1	*NUP133*	Mouse embryos	Loss of function mutant *Nup133*^*merm*^	ES and epiblast cells remain undifferentiated despite stimuli for differentiation. No impact on NPC formation. Inefficient neural differentiation.	[152]
2	*NUP107*	Zebrafish embryos	Loss of function mutant Nup107^tsu068Gt^	Perturbed chondrogenic differentiation, impaired mRNA export, and apoptosis induction during skeletal development.	[153]
		*Drosophila*	Nup107^D364N^ (recessive missense mutation)	Defective oogenesis and female infertility.	[154]
3	*ELYS*	Zebrafish	Flotte lotte (*flo*) mutant (nonsense mutation, null mutation for *ELYS*)	Hinders differentiation of intestinal epithelium, unusual aggregates of NPC in the cytoplasm, and cells undergo apoptosis.	[155–157]
		Zebrafish	*flo* mutant	Impaired neurogenic divisions in retinal precursor cells prevent them from entering terminal neuronal differentiation stages.	[158]
		Mouse embryos	Primary null mutant blastocysts cell line	Abnormal differentiation with impaired inner cell mass outgrowth.	[159]
4	*SEH1*	*Drosophila*	seh1^Δ15^ null mutant	Defects during oocyte differentiation, formation of nurse cells, and oogenesis. Reduced fertility and delayed mitosis.	[160]
		Rat and mouse OPCs cell lines, mice	Seh1cKO mice, siRNA knockdown rat OPCs	Inhibits oligodendrocyte differentiation and causes defects in myelination. No effect on NPC assembly and transport function.	[146]
5	*SEC13*	Zebrafish	Sec13^sq198^ (frameshift mutation)	Impaired retinogenesis with disrupted retinal lamination and more apoptotic cells. Altered NPC formation, reduced expression of Nups, and disrupted mRNA export.	[161]
		Zebrafish	Knockdown	Impaired photoreceptor differentiation during retinal development.	[162]
6	*NUP98/96*	*Drosophila*	Nup98–96^2288^ (transposon insertion)	Defective and early onset of differentiation in spermatocytes. Reduced number of germline cells.	[163]
**C**	**Inner ring Nups**				
1	*NUP155*	Mouse ESCs	*nup155*^*+/-*^	Decrease in proliferation and pluripotency marks - Oct4, Sox2 and Nanog.	[164]
2	*NUP93*	Immortalized human podocytes	shRNA	Reduction in proliferation. Compromised NPC integrity.	[165]
		Primary human epidermal keratinocytes	shRNA	Progenitor cells lose regenerative capacity. NPC numbers and transport are unaltered. Upregulation of differentiation genes.	[143]
3	*NUP62*	Zebrafish	CRISPR/Cas9-dependent gene knockout	Disrupted chondrogenic differentiation. Activation of apoptosis and suppression of Wnt/β-catenin signaling.	[166]
**D**	**Cytoplasmic filaments**				
1	*NUP358/RANBP2*	C2C12 (myoblast cell line)	siRNA	Suppress muscle differentiation. Structural alterations in NPC.	[144]
		Cultured rat embryo hippocampal neurons	siRNA	Formation of multiple axons indicating faulty axon-dendrite differentiation.	[167]
2	*GLE1*	Zebrafish	gle1^hi4161a^ Insertional mutant (loss of function)	Apoptosis in neural precursors inhibits differentiation. Defect in Schwann cell precursor proliferation and differentiation.	[168,169]
3	*RAE1*	*Drosophila*	rae1^Z5584^ (G129D point mutation)	Postmeiotic spermatid differentiation arrested and no mature sperm in the testes.	[170]
**E**	**Nuclear Basket**				
1	*NUP50*	C2C12	shRNA	Impaired muscle differentiation and myotubes fail to attain multinucleate morphology.	[171]
2	*NUP153*	Mouse ESCs	shRNA	Premature neuroectoderm differentiation without alteration in pluripotent markers and nuclear transport function of NPC.	[150]
		Cultured rat & mouse hippocampal NeuPCs	shRNA	Induction of neural and astroglial differentiation.	[149]
		Mouse NSCs	siRNA	Reduced neurosphere formation during neural differentiation.	[172]
3	*TPR*	C2C12	shRNA	Prolonged proliferation and hindrance in differentiation.	[145]

## Transcriptional regulation by Nups during differentiation

**Figure 4.**
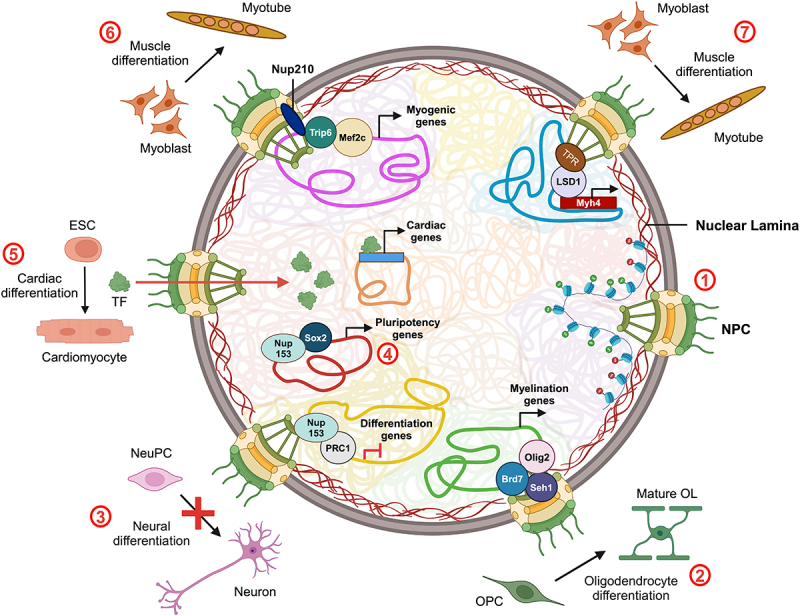
**Nucleoporins transcriptionally regulate differentiation.** Schematic representing the mechanisms by which NPCs modulate differentiation in multiple cell types. (1) NPCs are usually associated with open chromatin enriched with active histone modifications [149,175,176] (2) Seh1 recruits transcription factor, Olig2 which is involved in oligodendrocyte differentiation, and Brd7, a component of the SWI/SNF chromatin remodeling complex at promoters of neural differentiation genes to promote oligodendrocyte differentiation [146] (3) Nup153 also interacts with PRC1, an epigenetic regulator that silences developmental genes both at the nuclear periphery and nucleoplasm (not represented here) to inhibit neural differentiation [150] (4) Nup153 interacts with Sox2, a transcription factor both at the nuclear envelope and at the nuclear interior and induces expression of genes involved in maintaining pluripotency genes in NeuPCs [149] (5) Import of transcriptional factors such as Mef2c, Nkx2.5, and GATA4 enhanced during cardiac differentiation to promote expression of cardiac-specific genes. (6) In muscle differentiation, Nup210 associates with Trip6, a transcriptional co-regulator of Mef2c required for muscle differentiation and promotes myogenesis [177] (7) TPR recruits histone demethylase, LSD1 to the promoter of *MYH4* gene, critical for muscle differentiation and induces its transcription [145] OL (oligodendrocyte), OPCs (Oligodendrocyte Progenitor Cells), NeuPc (Neural Progenitor Cells), ESC (Embryonic Stem Cells), TF (Transcription Factors). (created with BioRender.com).

NPCs undergo dynamic remodeling and mediate nucleocytoplasmic communication to meet the increased demands of nuclear transport during differentiation. In *Xenopus laevis* oocytes, the number of NPC embedded in the nuclear envelope increases during later stages of oogenesis [173,174]. Nuclear pores are abundant in murine cardiomyocytes as compared to ESCs. Additionally, the pore diameter also increases upon differentiation of pluripotent stem cells into cardiomyocytes to enhance nuclear import of cardiomyocyte-specific transcription factors namely – Mef2c, Nkx2.5, and GATA4 (Figure 4) [148]. The diameter of the upper rim of the NPC also enlarges along with an increase in nuclear export function in differentiated C2C12 cells. It is possible that transcription factors enter the nucleus as a consequence of the increased diameter of the NPC. Contact mode Atomic Force Microscopy (AFM) revealed a plugged appearance of NPCs in differentiated myotubes than undifferentiated myoblasts [144]. This plugged morphology is due to the accumulation of ribonucleoproteins exiting the NPC, indicating their active export [174]. Depletion of nucleoporins such as Sec13 and Nup107 results in a reduced number of NPCs, which impairs its ability to transport cargo across the nuclear envelope along with defects in differentiation across cell types [153,161].

During apoptosis, caspases degrade certain Nups across different NPC subcomplex, namely – Nup358, Nup214, Nup153, Tpr, Nup50, Pom121, Nup93 and Nup96, which further abrogate NPC structure and increase the transport of pro-apoptotic proteins [178,179]. Interestingly, during muscle differentiation, partial degradation of Nups in the cytoplasmic filaments (Nup358 and Nup214) and nuclear basket (Nup153 and Tpr) by caspases impairs nuclear transport function. This altered and trimmed NPC overlaps with an increase in the nuclear accumulation of Focal Adhesion Kinase (FAK), which removes gene repression complex, NuRD (Nucleosome Remodeling and Deacetylation) complex from methylated CpGs in the promoter of *MYOG*. Hence, the trimming of the NPC inhibits the nuclear export of FAK and promotes the expression of myogenesis-associated genes [179]. In summary, architectural changes of the NPC facilitate transcription during the transition from pluripotency to specialized and differentiated cell types.

Additionally, NPCs modulate gene expression by regulating the spatial organization of the gene or by recruiting transcriptional regulators. NPCs usually maintain an open chromatin and a transcriptionally conducive microenvironment [175,180]. Active genes often relocate toward NPCs, enhancing their expression and promoting transcriptional memory [130]. Nucleoporins play an important role in chromatin decondensation and gene regulation during differentiation. Alternatively, certain Nups such as Nup93, Nup98, Nup155, and Nup153 are involved in transcriptional repression [181–183]. For instance, Nup93 associates with and represses the *HOXA* gene which is essential for differentiation and early development [184]. Furthermore, off-pore Nups such as Sec13, Nup98 and Nup153 also impact gene expression within the nuclear interior (Figure 4) [185,186]. The mechanisms by which Nups of each NPC sub-complex regulate differentiation are discussed in this review.

The cytoplasmic filament nucleoporin RAE1, along with Nup98, regulate genes involved in the maintenance of the epidermal progenitor state. RAE1, Nup98 and HDAC1 form a complex and bind to the TSS of *DNMT1* - an epigenetic repressor. DNMT1 is required for suppression of terminal differentiation. Therefore, the loss of any component in this complex results in premature differentiation of keratinocytes [187]. In addition, a mutation in RAE1 (rae1^Z5584^) shows defects in nuclear morphology and meiotic chromatin condensation in spermatocytes, leading to impaired spermatid differentiation [170]. Nup98 is also present in the inner ring of the NPC. It is abundant in terminally differentiated cardiomyocytes as compared to the progenitor cells [188]. Nup98 exists as on-pore and off-pore states and associates with the chromatin to induce expression of developmentally regulated genes [181]. How does Nup98 achieve such a remarkable function notwithstanding its localization at both the NPC and the nucleoplasm? At the nuclear pore, Nup98 associates with and activates *GPM6B, SOX5*, and *ERBB4* genes involved in the initial stages of stem cell differentiation, while at the nuclear interior, Nup98 enhances expression of *GRIK1, NRG1*, and *MAP2* genes required during later stages of neuronal differentiation in Neural Progenitor Cells (NeuPCs). These results suggest that Nup98 is associated with active chromatin. However, in terminally differentiated lung fibroblast cells, IMR90, Nup98 is associated with chromatin enriched in H3K9me3 - a repressive mark [181]. Therefore, Nup98 surprisingly shows a dual and potentially context-specific role in both induction and repression of genes.

Depletion of Nup155 (inner ring nucleoporin) decreases expression of pluripotency factors such as Oct4, Sox2 and Nanog in mouse ESCs, highlighting its role in maintaining stemness [164]. Similarly, Nup93 also maintains keratinocytes in an undifferentiated state. Loss of Nup93 increases the nuclear pool of NF-κB p65/p50 transcription factors, which induces early keratinocyte differentiation [143]. In pluripotent human embryonal carcinoma cells, Nup93 binds to and represses the *HOXA* gene cluster [184,189]. Active *HOXA* gene clusters are essential for early development and differentiation of osteoblasts, adipocytes, and thymocytes, among others [190,191]. In undifferentiated cells, Nup93 tethers the *HOXA* gene to the nuclear envelope and maintains HOXA in a poised state in conjunction with the chromatin insulator CTCF. ChIP-PCR data shows that Nup93 and CTCF bind to the 3’ and 5’ regions of the *HOXA* gene promoter, respectively. Upon induction of differentiation by RA, the occupancy of Nup93 on *HOXA* decreases and CTCF binding promotes untethering of *HOXA* from the nuclear periphery, resulting in its activation. In terminally differentiated neuronal cells, Nup93 enrichment is reestablished on the *HOXA* gene cluster along with its relocalization to the nuclear envelope and repression. Thus, Nup93 and CTCF temporally modulate the spatial organization and expression of the HOXA gene cluster during different stages of neuronal differentiation [189].

NDC1, POM121, and Nup210 are the three transmembrane nucleoporins that anchor NPC to the nuclear envelope [192]. Nup210 is incorporated in the NPCs during later stages of differentiation and is absent in undifferentiated murine myoblast cell line C2C12 and neural ESCs [151]. Similarly, in zebrafish embryos, Nup210 is required for muscle growth but not the initial muscle fiber formation. The C-terminal of Nup210 binds to Trip6 - a transcriptional co-regulator of Mef2c. This Mef2c-dependent transcriptional complex at NPC activates genes required for muscle differentiation (Figure 4). Pom121 also associates with Trip6 but is dispensable in the presence of Nup210 during myoblast differentiation [177]. Thus, NPCs function as scaffolds, facilitating the assembly of tissue-specific transcription complexes to regulate gene expression at the nuclear periphery.

Multiple Nups in the outer ring subcomplex of the NPC are essential for cell fate determination, especially in germline cells (Table 1). In *Drosophila*, Nup98/96 maintains the undifferentiated state of spermatocytes [163]. Additionally, the interaction of nucleoporin Seh1 and Mio protein is critical for maintaining nuclear structure and meiotic progression during the early stages of oogenesis in *Drosophila* [160]. Seh1 recruits Olig2 - a transcription factor involved in oligodendrocyte differentiation, and Brd7 - a component of the SWI/SNF chromatin remodeling complex at the NPC (Figure 4). Knockdown of Seh1 in oligodendrocytes disrupts this interaction and reduces promoter enrichment of Olig2, Brd7, and H3K27ac on myelination-associated genes. This results in chromatin condensation proximal to the promoters of differentiation and myelin-related genes, thereby repressing their transcription. Therefore, Seh1 promotes chromatin accessibility for the transcriptional machinery [146].

The nuclear basket has three off-pore Nups (Nup50, TPR and Nup153) that are often involved in the transcriptional regulation of genes both at the nuclear envelope and in the nucleoplasm. Nup50 associates with active chromatin that are enriched in the histone mark H3K4me3 and active RNA Pol II [193]. Knockdown of Nup50 in myoblast cell line C2C12 inhibits differentiation into myotubes [171]. But whether Nup50 is directly associated with myogenic genes to regulate their expression is yet to be deduced. However, TPR binds to myogenic genes, *MYH4* and *MEF2C* to induce their transcription during murine muscle differentiation. TPR also recruits Lysine-Specific Demethylase 1 (LSD1) to the promoter of *MYH4*, enhancing its accessibility for gene activation at the onset of differentiation (Figure 4) [145]. While Nup50 and TPR associate with open chromatin, Nup153 interacts with both open and condensed chromatin in a context-dependent manner. Nup153 shows a bimodal regulation wherein its occupancy at TSS of gene promoters induces their expression, but its enrichment at transcriptional end sites mediates gene silencing. Additionally, ChIP-Seq data reveals the association of both H3K4me3 (active) and H3K27me3 (inactive) histone marks on genes showing occupancy of Nup153, suggesting that Nup153 potentially maintains bivalent chromatin in a poised state [149]. Nup153 inhibits neural commitment and maintains pluripotency in undifferentiated cells by two potential mechanisms: (i) Nup153 mediates chromatin compaction through the recruitment of chromatin remodeler Polycomb Repressive Complex 1 (PRC1) to lineage-specific genes that leads to their repression [150] (ii) Nup153 associates with Sox2 to upregulate expression of pluripotency genes such as *YY1* (Figure 4) [149]. Therefore, knockdown of Nup153 leads to neural differentiation in mouse ESCs [150].

In summary, the NPC is required not only for nucleocytoplasmic transport but also for regulating gene expression during cellular differentiation. The precise organization and stoichiometry of nucleoporins within the NPC are critical for maintaining cellular functions and facilitating developmental and differentiation. In addition to undergoing structural remodeling, Nups regulate cell-fate decisions by precisely controlling nuclear transport of gene regulatory factors and by acting as scaffolds for transcriptional complexes to modulate transcriptional outcome. Furthermore, Nups directly associate with chromatin to dynamically activate or repress gene expression. The spatial organization of chromatin relative to NPCs is crucial for controlling gene expression programs that drive cell fate decisions during development. Overall, the dynamic interaction of chromatin with NPC provides insights into the differential roles of Nups across states of cell differentiation.

## Nuclear Envelopathies

The group of rare diseases caused by defects in the nuclear envelope are collectively referred to as envelopathies. Nuclear envelopathies are characterized by aberrant nuclear morphologies. Among these, mutations in lamin-encoding genes result in a broad but rare category of diseases referred to as laminopathies – majority of which have been linked to mutations in *LMNA* [194]. Laminopathies include Emery–Dreifuss Muscular Dystrophy (EDMD), Congenital Muscular Dystrophy (CMD), Dilated Cardiomyopathy (DCM), Limb-Girdle Muscular Dystrophy (LGMD), Hutchinson-Gilford Progeria Syndrome (HGPS) and Mandibuloacral Dysplasia (MADA) among others [195,196]. Furthermore, mutations in *LMNA* show cardiac fibrosis, which is manifested as dilated, arrhythmogenic, or non-compaction cardiomyopathies [197]. Mutations in *TOR1AIP1* gene encoding LAP1 are very rare, with only 23 cases reported worldwide. The majority of the cases are associated with consanguineous marriages and are typified with phenotypes including muscular dystrophy, congenital myasthenic syndrome, cardiomyopathy and multisystemic disease [198]. Additionally, mutations of NPC components manifest as tissue-specific pathologies in rare genetic disorders. Despite the presence of NPCs in all nucleated cells, most of these diseases specifically affect one or a few organs (reviewed in [199]). Clinical manifestations associated with disease-causing mutations in nuclear envelope proteins are summarized in Table 2. Currently, there is no cure for envelopathies and treatment options largely include symptomatic therapies and supportive care. Therapeutic limitations exist because envelopathies are caused by germline mutations. Some modes of treatment include (i) novel small-molecule inhibitors; for example, lonafarnib- a farnesyltransferase inhibitor is the first FDA-approved drug for HGPS that reduces the accumulation of progerin at the nuclear envelope [229], (ii) CRISPR-based gene editing, which allows correction of disease-causing mutations [230], (iii) gene replacement therapy, wherein a functional copy of the defective gene can be introduced using viral vectors, (iv) RNA-based therapies, which includes introduction of RNAi to downregulate the defective gene or synthetic mRNA encoding functional proteins. Some of the major bottlenecks of these treatment strategies include (i) challenges in effective delivery to target tissues such as muscle and heart (ii) permeability to the nucleus (iii) off-target effects on other critical genes (iv) ethical considerations. These challenges can be overcome by integrating advanced technologies such as nanotechnology and biology to precisely target affected tissues.

**Table 2.** Clinical manifestations associated with disease caused by mutations in nuclear envelope genes.

SI#	Gene		Disease	Clinical manifestation	Biological phenotype	Reference
**A**	**Nuclear Lamins**					
1	*LMNA*	Emery-Dreifuss Muscular Dystrophy (EDMD) (also caused by mutation in EMD, SYNE1/2 or SUN1/2)		Skeletal muscular weakness, joint contractures in early childhood, and cardiomyopathy with conduction abnormalities in adolescence	Defects in muscle differentiation and development	[200–204]
		Hutchinson- Gilford Progeria Syndrome (HGPS)		Accelerated aging, autonomic nervous system problems, head, narrow nasal features, fat loss, delayed primary teeth eruption, joint contractures, hearing loss, sclerotic dermis, bone dysplasia, lipodystrophy and cardiovascular defects	Impaired differentiation commitment during early development, including keratinocyte, chondrogenic, osteogenic and adipogenic differentiation, and premature senescence	[82,205–207,231]
		Mandibuloacral Dysplasia (MADA)		Early aging, lipodystrophy, skeletal abnormalities, craniofacial deformities, progressive acral osteolysis, and skin atrophy	Abnormal bone development with craniofacial anomalies	[208]
		Dunnigan-type familial partial lipodystrophy		Abnormal fat accumulation in the neck, face, and abdomen, and metabolic disorders including diabetes and liver steatosis	Defective adipocyte differentiation	[209]
		Congenital Muscular Dystrophy (CMD)		Muscle weakness prior to the attainment of ambulation and delayed motor milestones	Weak muscle tone and increased muscle atrophy	[20,73,210]
		LMNA-related Dilated Cardiomyopathy (DCM)		Cardiac symptoms like atrioventricular blocks, ventricular tachycardia and fatal sudden heart failure	Abnormal nuclear envelope morphology and differential euchromatin organization	[211–213]
		Charcot-Marie-Tooth Disease		Progressive foot deformities, sensory loss, weakness in the lower extremities	Abnormalities in Schwann cell differentiation.	[214]
2	*LMNB1*	Adult-onset Autosomal Dominant Leukodystrophy		Autonomic nervous system problems, cognitive impairment and muscle weakness	Increased nuclear envelope rigidity and demyelination	[215–217]
	*LMNB1 and LMNB2*	Microcephaly		Reduced head size, neurological deficits and intellectual disability	Neurodevelopmental delay	[218]
3	*LMNB2*	Progressive Myoclonus Epilepsy (PME) with early ataxia		Progressive neurological decline and seizures	Abnormal cortical development	[219]
**B**	**LINC Complex**					
1	*SYNE1*	Congenital Myopathy		Muscle weakness and delayed motor development	Nuclear deformations, impaired muscle differentiation	[20,73]
**C**	**Lamin B-receptor (LBR)**					
	***LBR***	Pelger-Huet Anomaly (PHA)		Skeletal anomalies and developmental delay	Aberrant chromatin organization, nuclear morphology and abnormal granulocyte nuclear differentiation	[220,221]
		Greenberg Dysplasia		Bone abnormalities in fetus and abnormal chondro-osseous calcification	Disturbed leukocyte development with abnormal chromatin structure	[222]
**D**	**LEM-domain proteins**					
1	*LEMD2*	LEMD2 ‐ associated progeroid syndrome		Growth retardation, skeletal, neuronal, and muscle abnormalities, and cerebellar intention tremor	Aberrant nuclear morphologies	[223]
2	*MAN1*	Osteopoikilosis, Melorheostosis and Buschke - Ollendorff syndrome		Increased bone density	TGF-β signaling altered during embryonic development and abnormal bone development	[119,224,225]
**E**	**NPC**					
1	*NUP85*	Autosomal Recessive Microcephaly (MCPH)		Hypotonia, congenital cataracts, microcephaly, aberrant brain imaging, and early death from respiratory failure	Defects in proliferation and/or differentiation of neural precursor cells.	[226,227]
2	*NUP107*	XX gonadal dysgenesis		Underdeveloped ovaries	Defective nurse cells and impaired oogenesis	[154]
3	*GLE1*	Lethal Congenital Contracture Syndrome 1 (LCCS1)		Total fetal immobility in the womb leading to death before birth	Motor neuron development defects and defects in proliferation and/or differentiation of Schwann cell precursors.	[168,169]
4	*NUP93*	Steroid Resistant Nephrotic Syndrome (SRNS)		Severe proteinuria	Reduced proliferation rate of human podocytes	[165]
5	*NUP188*	Sandestig-Stefanova syndrome		Microcephaly and congenital cataracts	Delayed myelination	[228]

## Conclusion and Perspectives

The nuclear envelope proteins play critical and multifaceted regulatory roles in differentiation and cell-fate decisions. This review highlights the intricate mechanisms by which major components of the nuclear envelope regulate nuclear architecture and spatio-temporal organization of chromatin to drive cell fate commitment. While condensed and repressive heterochromatin is organized at the nuclear lamina and enriched in LADs, chromatin organization at the nuclear periphery is heterogeneous [6]. This heterogeneity arises from differential chromatin organization at the nuclear lamina as compared to the NPC [232]. Although NPC and lamins are localized within ~30 nm, electron microscopy reveals that NPCs are largely associated with active, decondensed chromatin [233,234]. In contrast to the megadomains of LADs, Nups occupancy is limited to smaller regions of chromatin, particularly gene regulatory elements. Association with nuclear lamina typically results in gene repression, while interaction with NPCs can lead to either gene activation or repression [150,180,189].

LADs are rearranged during cell fate determination. Pluripotency genes associate, while lineage-specific genes dissociate from the nuclear lamina during differentiation [27]. Recent evidences reveal a nuanced organization of the LADs into two sub-types: (a) highly enriched in lamin B1, H3K9me2 and localized in compartment B; (b) intermediate occupancy of lamin B1 and H3K9me2 and is localized closer to compartment A [26,27]. Therefore, it is highly likely for genes in these LAD sub-types to be in a poised state, which facilitates their timely activation across stages of differentiation. We surmise that certain regions of chromatin associate with the A- or B-type lamins in a selective manner [235]. High-resolution STORM imaging reveals distinct but overlapping regions of A- and B-type lamin meshwork and therefore a potentially variable interaction of chromatin to form microdomains [236]. DamID and ChIP-Seq analysis of individual lamin isoforms in undifferentiated and differentiated cells are likely to reveal either similar or unique enrichment of A- and B-type lamins on chromatin. Of note, ChIP with lamin A/C antibody is likely to pull down lamin A/C fraction at the nuclear periphery as well as in the nucleoplasm (phosphorylated lamin A/C). Therefore, it is useful to include ChIP of pSer22 lamin A/C in order to determine the nucleoplasmic lamin A/C-chromatin fraction. Previously, ChIP-Seq of pSer22-Lamin A/C demonstrated its occupancy on active enhancers in the nucleoplasm [76]. Whereas fewer studies show the association of lamin A/C with both eu- and heterochromatin in the nucleoplasm. This suggests that the activate or repressive role of soluble lamin A/C in the nucleoplasm is context-dependent. Furthermore, lamin A/C in the nucleoplasm interacts with LAP2α via BAF. As discussed previously, LAP2α binds to the promoter sequence of myogenic genes and restricts the binding of soluble lamin A/C to these regions, thereby facilitating transcriptional activation of myogenic genes [52,107,108].

NPCs modulate gene expression by regulating the transport of transcription factors into the nucleus. For instance, the import of Mef2c – transcription factor involved in differentiation increases during cardiac differentiation [148]. Additionally, Nups assist in organization of lineage-specific genes and formation of transcriptional hubs at the TSS by interacting with transcription factors to promote gene expression [146,149]. As a case in point, nucleoporin TPR binds to *MEF2C* gene to organize it proximal to the nuclear envelope in undifferentiated cells and upon differentiation, the TPR-*MEF2C* interaction reduces, suggesting its release from the nuclear envelope [145]. While another nucleoporin, Nup210 forms a transcriptional complex with Mef2c protein along with the transcriptional co-activator Trip6 in differentiated myotubes [177]. Thus, Nups are not only involved in the spatial organization of *MEF2C* gene at the nuclear periphery but also in recruiting the protein in a Mef2c-dependent transcriptional complex at the NPC to create a transcriptionally favorable environment for the expression of lineage-determining genes.

In conclusion, while the cross-talk between nuclear envelope proteins and chromatin organization plays a key role in differentiation, the underlying mechanisms remain unclear. Insights into these mechanisms aim to unravel early prognostic markers and potential therapeutic targets for nuclear envelopathies. Further, combining multi-omics analysis of DamID, ChIP-Seq, RNA-Seq, and high-resolution microscopy or expansion microscopy of nuclear envelope proteins across cell types and diverse physiologically relevant mechanobiological platforms will enable in uncovering the precise regulatory mechanisms of gene expression and chromatin dynamics during cell differentiation.

## Data Availability

Data sharing is not applicable for this article as no new data were created or analyzed in this study.
